# Public opinion on alcohol policies in Sri Lanka

**DOI:** 10.3389/fpubh.2024.1409012

**Published:** 2024-07-12

**Authors:** Nadeeka K. Chandraratne, Nalin Singh Negi, Hasini Siyambalapitiya, Sampath De Seram, Nidarshana Selladurai, Karieshini Pieris, Rachel Rothenstein-Henry, Nandita Murukutla

**Affiliations:** ^1^Faculty of Medicine, University of Colombo, Colombo, Sri Lanka; ^2^Vital Strategies, New Delhi, India; ^3^Alcohol and Drug Information Centre, Colombo, Sri Lanka; ^4^Vital Strategies, New York, NY, United States

**Keywords:** alcohol, public opinion, tax, policy, Sri Lanka

## Abstract

**Background:**

Alcohol imposes a significant burden on health, social and economic systems in Sri Lanka. In the present economic crisis taxes on alcohol provides necessary revenue increases. Yet, the perception of the public on alcohol policies in Sri Lanka is not well explored.

**Objectives:**

This opinion survey was conducted with the aim to understand the public’s awareness on alcohol harm, alcohol industry influences, barriers and facilitators for implementing alcohol control policies in Sri Lanka, and the level of public support for alcohol policies, particularly taxes on alcohol products.

**Methods:**

A street intercept survey among 997 participants (with a ratio of 2:1 for males and females) selected through a cluster sampling method responded to an interviewer administered questionnaire. Bivariate and multivariate analyses were conducted to determine associations and a *p* < 0.05 was considered significant.

**Results:**

Among the respondents, 36.1% have consumed alcohol at least once in their lifetime and 29.1% have consumed alcohol during the past 12 months with a significant gender difference (females - 2.8%; males- 43.4%; *p* < 0.001). Significant proportions of both men (81.4%) and women (71.8%); *p* < 0.017 agreed that policy measures to reduce alcohol consumption would benefit the government including a significant proportion (73.8%, *p* < 0.008) of alcohol users. The vast majority −72.8%- agreed that increasing alcohol prices would help address the alcohol consumption problem in Sri Lanka. Moreover, only 30.8% of men and 44.3% of women agreed that the government’s alcohol laws are currently strong enough to protect people from alcohol harm. The regression analysis revealed that men are 2.43 times more in agreement with the statement that “policy measures aimed at reducing alcohol consumption can benefit the public” as compared to women. However, individuals aged 50–64 years are 40% less likely to agree with this statement as compared to 18–33 years.

**Conclusion:**

The majority of the public, including people who consume alcohol, are supportive of improving alcohol related policies, including taxes, and acknowledge negative impact of alcohol consumption on the country. This presents a clear opportunity for Sri Lanka to strengthen and enforce the alcohol related policies to protect and improve public health.

## 1 Introduction

Alcohol contributes significantly to premature mortality and disability globally, particularly due to alcohol-related diseases and injuries (1). Despite lower *per capita* alcohol consumption in lower-income countries, the burden of illness associated with alcohol is inversely correlated with national income levels. Notably, the same quantity of pure alcohol causes 3.7 times more harm in terms of alcohol-attributable disability-adjusted life years in low-income countries compared to high-income countries (1).

This trend is evident in Sri Lanka. Sri Lanka is a middle-income country situated in South Asia, with an officially estimated Gross Domestic Product (GDP) of $74.4 billion at the end of 2022 (2), and a population of 21,670,000 (3). Alcohol-related harm places a significant burden on Sri Lanka’s health system. Annually the deaths due to non-communicable diseases (NCDs) such as liver cirrhosis and cancer are estimated to be 2,880 and 646 death per 100,000 respectively, while due to road traffic crashes are around 675 per 100,000 (4). This translates to an economic loss of approximately USD 885.86 million in 2015, 1.07% of the GDP of that year. The direct cost of alcohol related disease conditions was USD 388.35 million, which was 44% of the total cost, while the indirect cost was USD 497.50 million, which was 66% of the total cost. Road Injury cost was the highest cost category among the conditions studied (5).

Consequently, urgent multisectoral action is needed to address alcohol consumption as a significant public health issue. With the heavy NCD burden in the country, NCDs were identified as an important issue in the National Health Policy of Sri Lanka published in 1996, while the Health Master Plan for 2007–2016 identified prevention and control of the NCDs as a vital priority. With the aim of combatting the NCD epidemic, Sri Lanka’s National Policy and Strategic Framework for Prevention and Control of Chronic NCDs (NCD Policy) was implemented in 2010. Furthermore, in 2016, Sri Lanka launched the National Multisectoral Action Plan for the Prevention and Control of NCDs 2016–2020 (NMAP 2016) (6). In keeping with these national NCD policies, the National policy on Alcohol Control was launched in 2016. This provides 10 priority policy areas for alcohol control in Sri Lanka, highlighting the importance of pricing and taxation with high community involvement (7).

Understanding the landscape of alcohol production and distribution is crucial to effectively implementing these policies. The alcohol industry in Sri Lanka consists of several companies that produce and distribute alcoholic beverages, with the most prominent being the Distilleries Company of Sri Lanka (DCSL), Lion Brewery PLC, and Heineken Lanka Limited. Among the various types of alcoholic beverages in Sri Lanka, arrack (a locally produced alcoholic beverage that is consumed by a majority of alcohol consumers in Sri Lanka, which contains 33% ethanol), holds the largest market share, while beer, bottled toddy (a locally brewed alcohol beverage from the sap of the coconut flower, with less than 5% alcohol) and locally-made foreign liquor is also produced in smaller quantities. Alcohol distribution in Sri Lanka occurs through various channels, including licensed liquor outlets (known as wine stores), liquor bars, and hotels. Both bars and hotels are licensed to distribute alcohol with bars offering alcoholic beverages for takeaway and hotels allowing on-premises consumption. Alcohol is also distributed at private functions such as parties, etc. (8). The illicit alcohol typically brewed on a small scale and referred to locally as “kassippu” occupies a minor segment of the market. According to studies conducted by the Alcohol and Drug Information Center (ADIC) and the Institute of Policy Studies (IPS), illicit alcohol accounts for less than 10% of the market share (9).

Despite the establishment of the National Authority on Tobacco and Alcohol (NATA) Act in 2006 in Sri Lanka, which prohibits the direct, indirect and surrogate promotion of alcohol, the alcohol industry continues to promote their products through various means such as social media and increasingly through various social events. Online alcohol delivery is recognized as a rapidly emerging industry strategy, in line with online purchases more generally, and has increased the availability of alcohol (4). According to the NATA Act Violation Analysis conducted by ADIC in 2022, nearly 1,200 social media posts and videos were found promoting alcohol consumption (9).

In addition to these challenges, Sri Lanka is currently confronted with an acute and unique crisis situation resulting in soaring inflation, depleting foreign reserves and rapidly depreciating currency (10). The GDP at the end of 2022 indicated a decline of 16% from the GDP of $88.5 billion in 2021 (2). This economic contraction had caused the nation to be declared as one of the highest inflated countries in the world (10). Therefore, at present, tax policies have gained considerable prominence in public discourse, media coverage and governmental deliberations.

Alcohol taxation has emerged as a pivotal strategy, given its potential to improve government revenues during this difficult economic phase. Moreover, the World Health Organization (WHO) emphasizes alcohol taxation and pricing policies as strategies among the most effective and cost-effective alcohol control measures (11). The International Monetary Fund (IMF) Country report 23/116 recommends a 20% increase in excise taxes for alcohol and tobacco products as a measure in advancing fiscal consolidation and strengthening institutions (12).

To implement tax policies as an effective alcohol control strategy, it is crucial to foster an understanding on public perception regarding the harms associated with alcohol consumption and the economic significance of alcohol taxes, to fight against industry strategies that undermine alcohol control policies. Disseminating information through mass media is a cost-effective, population-level strategy which can be used to reach a larger population group with effective messages (13). Despite *ad-hoc* messages regarding alcohol harm displayed across a few media channels, currently, Sri Lanka lacks a national media strategy dedicated to publicizing accurate information regarding the severity of alcohol-related harms.

As an initial step toward reinforcing evidence-based alcohol control policies in Sri Lanka, the Alcohol and Drug Information Center (ADIC), Sri Lanka, as part of the RESET Alcohol initiative, conducted a public opinion survey to determine the public’s knowledge, attitudes and policy concerns on alcohol consumption, taxations and alcohol industry interferences. The RESET Alcohol initiative works with the aim of decreasing alcohol-related harms in hard-hit select countries. Funded by Open Philanthropy and led by Vital Strategies, RESET is a global consortium with Movendi International, Johns Hopkins University’s Tobacconomics team, Global Alcohol Policy Alliance (GAPA), NCD Alliance, and the World Health Organization (WHO).

With the combined collective force of national governments, civil society, research organizations, and global leaders in public health and alcohol policy, RESET Alcohol aims to develop and implement the three “best buy” evidence-based alcohol policies from the World Health Organization’s SAFER technical package (11). Its primary policy focus is increasing alcohol taxation, since elevated prices result in reduced alcohol consumption among consumers, consequently leading to fewer road fatalities, liver, heart and cancer deaths, domestic violence and other harms associated with alcohol use. Government partners are also encouraged to complement alcohol tax increases by addressing alcohol marketing and availability (11).

This collaboration is focused on strengthening the groundwork for implementing a comprehensive national media campaign to address this pressing public health concern. The opinion survey was carried out with the following objectives (1): to assess the public’s awareness on the alcohol industry’s influence on alcohol policy (2); to determine the public’s knowledge of alcohol harm and the barriers and facilitators of implementing alcohol policies; and (3) to identify the level of support from the public on alcohol policies.

## 2 Methods

### 2.1 Study design

This cross-sectional study utilized a street intercept survey methodology to collect data. While the survey targeted diverse districts and upheld a 2:1 ratio of male to female respondents, it is important to recognize that the sample’s representativeness of the entire country’s population may be limited.

### 2.2 Sampling method, sample size and data collection

Sri Lanka has nine provinces with each province consisting of two or more districts. In addition, there are three main socioeconomic sectors; urban, rural and estate. The study sample was selected by a multistage cluster sampling method to represent all provinces as well as the socioeconomic sectors. At the first stage of sampling five out of nine provinces were selected. Subsequently, at second stage, one district was purposively selected from each province resulting in the selection of five districts (Colombo, Badulla, Matale, Hambanthota and Jaffna) for the study representing the diverse sociocultural and economic background of the country. A quota of 200 was allocated to each district making the total sample size 1,000. From each district, 10 Grama Niladhari (GN) divisions–the smallest administrative division in Sri Lanka–were randomly selected. Each GN division was considered as a cluster and 20 interviews were conducted in each GN.

The 10 clusters from each district were: three clusters from bus stops/train stations/streets close to bus or train stations; two clusters from cafés or restaurants; three clusters from malls/grocery stores/supermarkets/markets; and two clusters a from religious places/recreational centers, to have a better representation of all socioeconomic strata. The interviewer approached every fifth person in a queue or after each successful interview at bus or train stations, or while entering a shopping mall, grocery store or park, or placing orders at a café or restaurant.

The survey was conducted by ADIC during the period from 14th June to 4th July 2023. Although there is no obvious seasonal variation in alcohol consumption in Sri Lanka, the consumption patterns are quite high during the weekends. To capture this variation, in the first week, data was collected on Monday, Thursday, and Saturday from 7 am to 1 pm, and in the second week, data was collected on Tuesday, Friday, and Sunday from 1 pm to 7 pm.

As we were keen to capture drinkers’ opinions on alcohol policies, the survey team decided to interview more males than females for the survey. It was decided to have a ratio of 2:1 for males to females, as the alcohol consumption among females in Sri Lanka is significantly lower (14).

The survey team interviewed 1,000 eligible individuals and 03 individuals had to stop the survey at the middle as they had to catch their busses, and therefore the total sample was 997. Although the affiliations for tobacco and alcohol industries were questioned as eligibility criteria, none of the participants declared it.

### 2.3 Measures

A pretested questionnaire was used to collect data, with an average completion time ranging from 30 to 40 min per respondent. The questionnaires were available in both local languages (Sinhalese and Tamil) to improve validity. The study collected quantitative data using closed-ended questions and some open-ended questions. The questionnaire comprised four parts: sociodemographic characteristics, alcohol use status, attitudes and opinions, and exposure to alcohol advertisements. The first section of the questionnaire initiated with cluster identification, encompassing a total of 21 questions. The initial question (Q01) sought general information to ascertain participants’ affiliations with alcohol or tobacco industries or market research, ensuring data integrity throughout the study. The alcohol use status questions were adopted based on the WHO STEPS survey questionnaire.

The attitudes and opinions section consisted of questions on attitudes toward alcohol use of the respondents as well as by others, the opinion questions focused on government policies toward the alcohol industry and marketing, and the efficient use of government revenue. The responses were given on a five-point Likert scale. Subsequent questions (Q02-Q10) were designed to assess participants’ knowledge and attitudes regarding policies and government actions related to alcohol consumption. Question 04 specifically examined participants’ awareness of diseases associated with alcohol consumption, providing nine predefined options and an open-ended response alternative. Responses were categorized into four levels to effectively gage participants’ knowledge levels. Question 05 explored participants’ opinions on various statements concerning alcohol policies, employing a Likert scale ranging from “Strongly Disagree” to “Strongly Agree,” including a “Do not Know” option. This facilitated a nuanced understanding of participants’ perspectives on policy measures and their implications. Question 06 investigated respondents’ perceptions of the government’s role in addressing alcohol consumption in Sri Lanka, presenting three statements with corresponding Likert-scale responses.

Question 07 evaluated participants’ support levels for potential government policies aimed at reducing alcohol consumption, presenting three statements for assessment via a Likert scale. Question 08 examined participants’ attitudes toward the allocation of tax revenue from alcohol for public programs. It offered six predefined public program options and included an open-ended alternative, alongside three levels of support: “Not increase at all,” “Increase a little,” and “Increase a LOT.” Question 09 presented participants with five policy options aimed at reducing alcohol consumption in Sri Lanka, evaluating their levels of support through a structured response format.

Additionally, the questionnaire included three questions focused on the unprompted recall of non-commercial advertisements related to the harms of alcohol (Q11-Q14), as well as demographic information (Q15-Q17) to contextualize respondents’ backgrounds concerning alcohol consumption.

### 2.4 Data analysis

Descriptive, bivariate and multivariate analysis was carried out using unweighted data in SPSS version 27.0. A chi-square test was applied to identify the associations between dependent variables (alcohol use status, attitudes, and opinions) and independent variables (gender, age, education, alcohol consumption in the previous 12 months, and exposure to alcohol-related media messages). A *p*-value of equal or less than 0.05 was considered significant for the chi-square test. Logistic regression analyses were conducted to measure the impact of independent variables on attitude toward alcohol, alcohol industry and governments policies toward alcohol and support for government policies toward alcohol. Socio demographic data, alcohol consumption in the past 12 months, and exposure to alcohol-related media messages served as independent variables. For the regression analysis on attitudes where the responses were on a Likert “Strongly Agree” and “Somewhat Agree” were combined and considered as “Agree” with a code of “1.” Conversely, responses of “Strongly Disagree,” “Somewhat Disagree,” and “Neither Agree nor Disagree” were grouped together and coded as “0.” For the regression analysis on support for government policies, where the responses were on a Likert scale “A great deal” and “very much” were combined and with a code of “1.” Conversely, all other responses were grouped together and coded as “0.” The results of the regression are presented as odds ratios.

### 2.5 Ethical and administrative requirements

The data collection was conducted in accordance with the Declaration of Helsinki. Before initiating the interview, the study was explained to all the participants, including the purpose of study, its importance, incentives and data security measures. Verbal consent was taken from each interviewee regarding their participation in the study. The questionnaire was administered only to those participants who agreed to participate. No personal identifiable information was obtained during the data collection.

## 3 Results

### 3.1 Sample characteristics

Table 1 represents the demographic characteristics of the survey participants. The study encompassed a diverse set of participants, capturing all social classes within the country. Nine hundred and ninety-seven (*n* = 997) individuals participated in the study. Of the study population, none of the respondents declared any links to the alcohol industry or to research or advertising related to the alcohol industry and therefore, no one was considered ineligible. In keeping with our 2:1 male to female selection criteria, 64.7% were men and 35.3% were women.

**Table 1:** Demographic characteristics of survey participants.

Question	Total	Alcohol consumption in past 12 months			Exposure to alcohol-related media messages		
		Yes	No	*P*-value	Aware	Unaware	*P*-Value
Gender				0.000*			0.057
Women	35.3	3.4	48.4		39.4	33.3	
Men	64.7	96.6	51.6		60.6	66.7	
Age				0.279			0.004*
18 to 33 years	38.5	34.5	40.2		44.5	35.5	
34 to 49 years	29.4	31.0	28.7		25.5	31.3	
50 to 64 years	21.8	22.1	21.6		17.6	23.8	
65+ years	10.3	12.4	9.5		12.4	9.3	
Highest level of education completed				0.066			0.000*
No formal schooling or primary education	9.9	12.8	8.8		5.8	12.0	
Studied or passed O/Ls	38.8	40.7	38.0		31.5	42.4	
Studied or passed A/Ls or higher	51.3	46.6	53.2		62.7	45.6	
Total sample	997	290	707		330	667	

*p ≤ 0.05.

The largest demographic group, comprising 38.5%, fell within the age category 18–33 years, while the majority comprising 51.3% had completed their education up to advanced level (A/Ls)^1^ or higher.

### 3.2 Alcohol consumption among survey participants

Table 2 reveals that among the study participants, 36.1% have consumed alcohol at least once in their lifetime and 29.1% have consumed alcohol during the past 12 months. Analysis of alcohol consumption in the past 12 months prior to the survey revealed a significant difference in relation to the gender of the study participants, with only 2.8% of the surveyed women and 43.4% of the surveyed men (*p* < 0.001) having reported that they have consumed alcohol within the preceding 12 months. Table 2 further presents the alcohol consumption by the survey participants based on their education level and exposure to alcohol-related media messages. Highest alcohol consumption ever (54.5%), was reported by respondents with no formal schooling or primary education, while 37.4% have consumed alcohol in the past 12 months. It is noteworthy that alcohol consumption was less among the participants with high education levels, denoting a decline in alcohol consumption with increased education levels. Only 38.2% of the participants with education completed up to O/L reported they have consumed alcohol at some point in their life, while only 30.9% who have completed their education up to advanced level reported that they have ever consumed alcohol. However, upon examining the education backgrounds of participants who reported alcohol consumption within the preceding 12 months, respondents with the highest education levels notably displayed a preference for beer (80.4%) over other types of alcohol, while toddy was mostly consumed by respondents with the lowest education levels (25.9%).

**Table 2:** Alcohol use by demographic characteristics of survey participants.

Alcohol consumption	Total	Gender (Baseline)			Education				Exposure to alcohol-related media messages		
		Women	Men	*P*-value	No formal schooling or primary education	Studied or passed O/Ls	Studied or passed A/Ls or higher	*P*-value	Aware	Unaware	*P*-value
Ever consumed alcohol	36.1	4.0	53.6	0.000*	54.5	38.2	30.9	0.000*	37.9	35.2	0.413
Consumed alcohol within the past 12 months	29.1	2.8	43.4	0.000*	37.4	30.5	26.4	0.066	32.4	27.4	0.103
Total sample	997	352	645		99	387	511		330		
Type of alcohol consumed											
Beer	74.4	50	75.4	0.032*	61.1	73	80.4	0.017*	80.0	71.5	0.078
Arrack	75.0	50	76	0.028*	81.5	71.6	75.9	0.335	77.6	73.6	0.406
Toddy	10.8	0	11.3	0.183	25.9	10.1	6.3	0.001*	10.4	11.1	0.847
Kasippu	4.7	0	4.9	0.396	7.4	6.1	2.5	0.206	4.8	4.7	0.960
Total sample	360	14	346		54	148	158		125	235	

**p ≤ 0.05. Sample for female is less than 30. So interpretation need to be carefully done.

**Figure 1:**
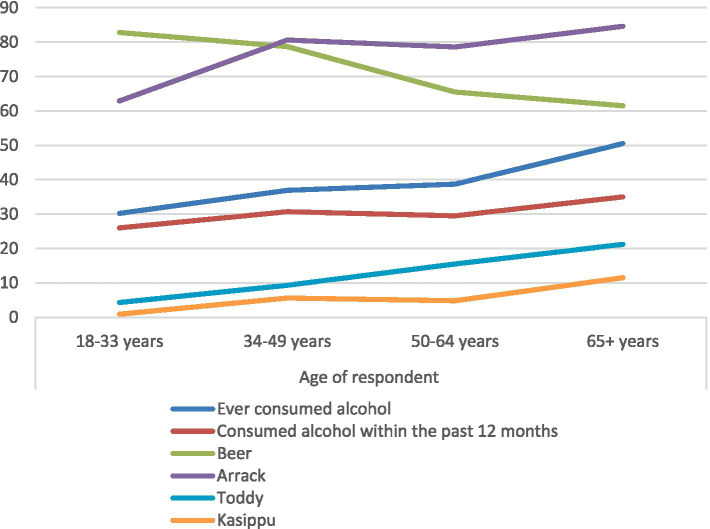
Drinking pattern of different age groups.

Figure 1 depicts the alcohol consumption in different age groups. The survey participants who have consumed alcohol in the past 12 months prior to the survey were asked to specify the types of alcohol they consumed, and they were given the option to provide multiple responses. The findings revealed that the consumption of all types of alcohol was higher among individuals above 65 years, where consumption of arrack, beer, toddy and kasippu amounted to 75, 74.4, 10.8 and 4.7%, respectively. Arrack was the most consumed alcohol type (75%), with notably low levels of consumption observed for toddy (10.8%) and kasippu (4.7%). The consumption of beer is highest among the younger age groups and shows a declining trend with age, while all the other types of alcohol types show an increasing trend with age. Alcohol consumption was significantly higher among respondents above 65 years of age, amounting to 50.5% (n = 103).

### 3.3 Attitude toward government and policy support

The participants were asked about their attitudes regarding alcohol consumption, the alcohol industry and government policies. The responses were analyzed according to the sociodemographic characteristics and also on their alcohol consumption and exposure to alcohol-related media messages. Tables 3–6 presents the subgroup analysis of participants according to the age, gender, education level, alcohol consumption status and the exposure to media messages. The chi square tests were done for each subgroup to determine the significance between the proportion that agreed compared to not agreed taken as the baseline.

**Table 3:** Association of attitude toward alcohol, alcohol industry and governments policies toward alcohol by demographic characteristics.

Statements	Total	Age of respondent					Gender			Education			
		18–33 years	34–49 years	50–64 years	65+ years	*P*-value	Women	Men	*P*-value	No formal schooling or primary education	Studied or passed O/Ls	Studied or passed A/Ls or higher	*P*-value
Attitude toward government													
It is important for my government to be involved in solving the problem of alcohol consumption in my country. (agree)	93.0	91.7	92.5	95.9	93.3	0.252	92.0	93.5	0.236	93.9	94.0	92.0	0.396
The government’s alcohol laws are currently strong enough and protect people from alcohol harms. (agree)	35.6	32.8	34.5	36.9	46.6	0.055	44.3	30.8	0.000*	26.3	39.5	34.4	0.002*
Support for government policies													
Policy measures to reduce the consumption of alcohol can benefit the public whether they consume alcohol or not. (agree)	78.0	78.7	79.1	75.6	77.7	0.952	71.8	81.4	0.017*	81.8	83.8	73	0.005*
Increasing the price of alcohol would help address the problem of alcohol consumption in Sri Lanka. (agree)	72.8	73.2	70	74.2	76.7	0.081	74.7	71.8	0.279	75.7	78.1	68.3	0.017*
Taxes on alcohol products are an effective way to reduce alcohol consumption. (agree)	75.3	73.9	75.1	79.8	70.9	0.349	75.6	75.0	0.530	74.7	80.9	71	0.000*
Attitude toward alcohol industry													
The alcohol industry is an obstacle to getting strong alcohol laws in the country. (agree)	60.3	60.4	60.4	60.9	59.3	0.854	62.5	59.2	0.818	54.6	63.1	59.5	0.000*
Attitude toward alcohol													
Alcohol is an obstacle to development. (agree)	78.3	75	78.5	79.7	87.4	0.115	84.4	75.1	0.010*	72.8	79.3	78.6	0.011*
Alcohol use can result in alcohol addiction. (agree)	84.5	82.8	82.6	85.3	95.2	0.136	90.0	81.5	0.003*	83.9	87.9	82.2	0.017*
Alcohol consumption affects others and the wider community negatively. (agree)	94.5	94	94.2	95	96.1	0.790	97.2	93.1	0.032*	94	95.1	94.1	0.015*
Drinking alcohol, a couple of times a week is not harmful to health. (agree)	66.1	67.7	65.5	64.0	66.1	0.513	71.6	63.1	0.002*	51.5	65.7	69.3	0.003*
Other statements													
Exposure to alcohol advertising encourages youth to initiate alcohol use. (agree)	89.2	88.1	87.7	91.3	94.2	0.313	92.3	87.6	0.133	85.9	91.2	88.5	0.001*

*p ≤ 0.05.

**Table 4:** Association of attitude toward alcohol, alcohol industry and governments policies toward alcohol by alcohol consumption and exposure to media campaigns.

Statements	Total	Alcohol consumption in past 12 months			Exposure to alcohol-related media messages		
		Yes	No	*P*-value	Aware	Unaware	*P*-value
Support for government policies							
Policy measures to reduce the consumption of alcohol can benefit the public whether they consume alcohol or not. (agree)	78.0	73.8	79.8	0.008*	67.3	83.4	0.000*
Increasing the price of alcohol would help address the problem of alcohol consumption in Sri Lanka. (agree)	72.8	67.0	74.0	0.005*	67.3	75.6	0.006*
Taxes on alcohol products are an effective way to reduce alcohol consumption. (agree)	75.3	75.5	75.1	0.110	72.7	76.5	0.199
Attitude toward government							
It is important for my government to be involved in solving the problem of alcohol consumption in my country. (agree)	93.0	91.8	93.5	0.534	91.5	93.7	0.203
The government’s alcohol laws are currently strong enough and protect people from alcohol harms. (agree)	35.6	36.9	35.1	0.078	25.5	27.7	0.445
Attitude toward alcohol industry							
The alcohol industry is an obstacle to getting strong alcohol laws in the country. (agree)	60.3	57.3	61.6	0.000*	56.1	62.5	0.050*
Attitude toward alcohol							
Alcohol is an obstacle to development. (agree)	78.3	69.6	81.9	0.000*	78.8	78.1	0.234
Alcohol use can result in alcohol addiction. (agree)	84.5	72.1	89.7	0.000*	84.5	84.6	0.076
Alcohol consumption affects others and the wider community negatively. (agree)	94.5	89.6	96.5	0.000*	95.2	94.2	0.664
Drinking alcohol, a couple of times a week is not harmful to health. (agree)	66.1	61.7	67.9	0.082	72.4	63.0	0.001*
Other statements							
Exposure to alcohol advertising encourages youth to initiate alcohol use. (agree)	89.2	83.5	91.6	0.000*	90.3	88.8	0.381

*p ≤ 0.05.

**Table 5:** Support for government policies related to alcohol by sociodemographic characteristics.

	Total	Age of respondent					Gender			Education			
		18–33 years	34–49 years	50–64 years	65+ years	*P*-value	Women	Men	*P*-Value	No formal schooling or primary education	Studied or passed O/Ls	Studied or passed A/Ls or higher	*P*-value
To what extent, would you support the government in implementing below policies?													
Stop indirect/direct alcohol advertising and promotion on traditional media, like TV, radio and social media. (a great deal + very much)	68.4	61.5	71.7	79.7	61.2	0.000*	62.8	71.5	0.001*	78.8	75.9	60.6	0.000*
Reduce the number of places and times where people can buy and consume alcohol. (a great deal + very much)	60.1	58.4	59.7	66.8	54.3	0.000*	57.1	61.8	0.066*	76.8	61.7	55.8	0.000*
Educate the population about alcohol harms. (a great deal + very much)	71.8	65.9	73.7	82.5	66.0	0.000*	65.6	75.2	0.000*	77.8	80.2	64.4	0.000*
Provide services for people affected by alcohol. (a great deal + very much)	70.2	65.9	72.7	76.5	66.1	0.000*	65.6	72.7	0.000*	77.8	75.5	64.7	0.000*
Raising the legal drinking age. (a great deal + very much)	66.1	59.7	65.5	76.0	70.9	0.000*	65.4	66.5	0.002*	80.7	75.7	55.9	0.000*
Any other recommendation for the government regarding controlling alcohol problems													
Cancelation of all alcohol licenses	25.9	22.4	25.9	31.3	27.2	0.117	24.7	26.5	0.536	28.3	29.5	22.7	0.062
Stop manufacturing, importing, and selling alcohol	36.5	37.5	31.4	41.5	36.9	0.123	33.5	38.1	0.148	42.4	38.2	34.1	0.189
Strengthen the law enforcement in the country	48.0	48.4	48.1	46.5	49.5	0.958	51.1	46.4	0.149	49.5	40.6	53.4	0.001*
Stop issuing new license to sell alcohol	15.6	15.4	14.7	18.9	12.6	0.444	16.8	15.0	0.474	10.1	17.1	15.7	0.236
Stop all types of alcohol promotions	12.0	11.2	12.3	12.0	14.6	0.827	10.5	12.9	0.274	7.1	11.6	13.3	0.207
Stop the alcohol industry’s influence on policy makers and politicians	3.8	3.6	3.1	3.2	7.8	0.167	4.5	3.4	0.371	2.0	4.4	3.7	0.539
Stop giving bar license to politicians	11.5	10.9	11.6	14.7	6.8	0.204	12.8	10.9	0.362	8.1	8.0	14.9	0.003*
Cancel bar license owned by politicians	8.9	7.8	8.9	11.1	8.7	0.614	9.4	8.7	0.714	5.1	5.9	11.9	0.003*
Total sample	997	384	293	217	103		352	645		99	387	511	

*p ≤ 0.05.

**Table 6:** Support for government policies related to alcohol by alcohol consumption status and exposure to media campaigns.

	Alcohol consumption in past 12 months			Exposure to alcohol-related media messages		
	Yes	No	*P*-value	Aware	Unaware	*P*-value
To what extent, would you support the government in implementing below policies?						
Stop indirect/direct alcohol advertising and promotion on traditional media, like TV, radio and social media. (a great deal + very much)	62.8	70.8	0.009*	53.3	75.9	0.000*
Reduce the number of places and times where people can buy and consume alcohol. (a great deal + very much)	45.5	66.2	0.000*	49.4	65.5	0.000*
Educate the population about alcohol harms. (a great deal + very much)	71.3	72.0	0.018*	58.2	78.6	0.000*
Provide services for people affected by alcohol. (a great deal + very much)	68.0	71.1	0.098	57.9	76.3	0.000*
Raising the legal drinking age. (a great deal + very much)	55.5	70.4	0.000*	55.5	71.4	0.000*
Any other recommendation for the government regarding controlling alcohol problems						
Cancelation of all alcohol licenses	19.7	28.4	0.004*	22.4	27.6	0.080
Stop manufacturing, importing, and selling alcohol	30.7	38.9	0.015*	32.1	38.7	0.043*
Strengthen the law enforcement in the country	47.9	48.1	0.963	57.3	43.5	0.000*
Stop issuing new license to sell alcohol	13.4	16.5	0.221	17.9	14.5	0.172
Stop all types of alcohol promotions	11.0	12.4	0.534	11.2	12.4	0.574
Stop the alcohol industry’s influence on policy makers and politicians	2.8	4.2	0.266	3.3	4.0	0.579
Stop giving bar license to politicians	13.4	10.7	0.226	14.5	10.0	0.036*
Cancel bar license owned by politicians	11.4	7.9	0.082	11.2	7.8	0.075
Total sample	290	707		330	667	

*p ≤ 0.05.

Seventy eight percent of survey participants, regardless of whether they consume alcohol, agree that policy measures to reduce alcohol consumption can benefit the public. While examining by gender significant proportions of both men (81.4%) and women (71.8%); *p* = 0.017 agree that policy measures to reduce alcohol consumption can benefit the public irrespective of their alcohol consumption status. A significant proportion (73.8%, *p* = 0.008) of alcohol users support this statement.

Moreover, only 30.8% men and 44.3% women agreed that the government’s alcohol laws are currently strong enough and protect people from alcohol harm. Of the total sample, 72.8% further agreed that increasing alcohol prices would help address the alcohol consumption problem in Sri Lanka.

Even among respondents with no exposure to media messages related to alcohol, 83.4% (*p* < 0.001) who did not report exposure to such media content agreed that policy measures to reduce alcohol consumption can benefit the public, while 75.6% (*p* = 0.006) also agreed that increasing alcohol prices would help address the alcohol consumption problem in Sri Lanka. More than half of the alcohol consuming survey participants (57.3%) agreed that the alcohol industry is an obstacle for the implementation of strong alcohol laws in the country, while 69.6% of the alcohol users agreed on the fact that alcohol is an obstacle to development. A significant proportion of the alcohol consuming respondents (83.5%, *p* < 0.001) also agreed that exposure to alcohol advertising encourages youth to initiate alcohol use.

### 3.4 Support for alcohol related policies

The respondents were given a set of five policy directives and asked about their support for each directive, with responses given on a Likert scale.

According to Tables 5, 6, significant support for government action regarding alcohol control policies was evident, with 68.4% (*p* < 0.001) of the respondents reporting high level of support for the government to stop both direct and indirect promotions of alcohol on all media platforms. Additionally, 60.1% reported high support for reducing the number of places and times where alcohol can be bought and consumed, while 71.8% supported public education on alcohol harms. Significant proportions of alcohol users also reported great level of support for the statements, amounting to 62.8% (*p* = 0.009), 45.5% (*p* < 0.001) and 71.3% (*p* = 0.018) respectively. Furthermore, 70.2% of the total survey participants supported providing services for people affected by alcohol, and 66.1% reported supporting raising the legal age for alcohol use. Moreover, even respondents without exposure to awareness messages on alcohol harm through media, reported significant support from the respondents for all statements with regard to government action for alcohol control policies.

### 3.5 Recommendations for policy actions

The participants were further asked about recommendations for government actions to address alcohol-related issues with the option to provide multiple responses. A significant majority across all education levels agreed on strengthening the law enforcement in the country (*p* = 0.001), while the second and third most significant suggestions were reported as stopping the provision of licenses to open liquor shops to politicians (*p* = 0.003) and canceling the licenses owned by politicians (*p* = 0.003) respectively. Among the respondents who had no exposure to media messages on alcohol harm, a significant proportion amounting to 43.5% (*p* < 0.001) also reported their support for strengthening the law enforcement in the country for alcohol control.

### 3.6 Methods of spending revenue generated from increased alcohol taxes

The survey participants were also questioned about their level of support for government policies on alcohol if the revenue generated went to various public programs including primary health care, primary education, public health education, safe roads, support for poor people and infrastructural development of the country (Tables 7, 8). The vast majority (73.2%) reported that their support for alcohol taxes would increase a lot if the revenues were used for programs to support the poor people, while the second highest proportion of the respondents (71.4%) mentioned increased support for infrastructural development of the country. The same level of support was reported by the respondents who consumed alcohol. as well, regarding the development programs. When asked about their level of agreement on proposed methods of spending the increased revenue, public health programs and programs to support poor people were among the highest rated (Tables 7, 8).

**Table 7:** Agreement on proposed methods of spending the increased revenue from alcohol tax by sociodemographic characteristics.

	Total	Age of respondent					Gender			Education			
		18–33 years	34–49 years	50–64 years	65+ years	*P*-value	Women	Men	*P*-value	No formal schooling or primary education	Studied or passed O/Ls	Studied or passed A/Ls or higher	*P*-value
Improve primary health care for all citizens (universal health care)						0.497			0.055				0.747
Not increase at all	7.3	6.8	7.8	7.8	6.8		6.0	8.1		8.1	6.7	7.6	
Increase a little	24.3	26.3	20.5	23.0	30.1		28.4	22.0		22.2	22.7	25.8	
Increase a lot	68.4	66.9	71.7	69.1	63.1		65.6	69.9		69.7	70.5	66.5	
Primary school education						0.631			0.780				0.092
Not increase at all	7.4	7.0	9.2	6.5	5.8		6.8	7.8		6.1	4.9	9.6	
Increase a little	22.0	24.2	20.8	19.4	22.3		21.3	22.3		19.2	23.3	21.5	
Increase a lot	70.6	68.8	70.0	74.2	71.8		71.9	69.9		74.7	71.8	68.9	
Public health education programs						0.935			0.454				0.170
Not increase at all	8.8	8.3	9.2	9.7	7.8		7.4	9.6		8.1	6.5	10.8	
Increase a little	22.3	23.2	20.1	22.1	25.2		21.9	22.5		18.2	23.8	21.9	
Increase a lot	68.9	68.5	70.6	68.2	67.0		70.7	67.9		73.7	69.8	67.3	
Safer roads for public						0.563			0.883				0.160
Not increase at all	13.3	13.5	14.3	14.7	6.8		13.6	13.2		10.1	12.1	14.9	
Increase a little	19.8	19.8	18.8	20.7	20.4		20.5	19.4		21.2	23.0	17.0	
Increase a lot	66.9	66.7	66.9	64.5	72.8		65.9	67.4		68.7	64.9	68.1	
Programs to support the poor, e.g., housing						0.704			0.073				0.093
Not increase at all	9.3	9.1	10.2	8.8	8.7		7.7	10.2		7.1	7.0	11.5	
Increase a little	17.5	19.0	16.7	18.4	11.7		14.8	18.9		14.1	17.1	18.4	
Increase a lot	73.2	71.9	73.0	72.8	79.6		77.6	70.9		78.8	76.0	70.1	
Infrastructural development of the country						0.881			0.391				0.236
Not increase at all	1	9.4	9.2	10.1	7.8		7.7	10.2		8.1	7.0	11.4	
Increase a little	19.3	19.8	19.8	19.8	14.6		20.2	18.8		21.2	20.2	18.2	
Increase a lot	71.4	70.8	71.0	70.0	77.7		72.2	71.0		70.7	72.9	70.5	
Total sample	997	384	293	217	103		352	645		99	387	511	

**Table 8:** Agreement on proposed methods of spending the increased revenue of alcohol tax by alcohol consumption status and exposure to media campaigns.

	Alcohol consumption in past 12 months			Exposure to alcohol-related media messages		
	Yes	No	*P*-value	Aware	Unaware	*P*-value
Improve primary health care for all citizens (universal health care).			0.162			0.011*
Not increase at all	9.7	6.4		7.3	7.3	
Increase a little	22.4	25.0		30.0	21.4	
Increase a lot	67.9	68.6		62.7	71.2	
Primary school education			0.060			0.666
Not increase at all	10.3	6.2		8.5	6.9	
Increase a little	22.8	21.6		21.8	22.0	
Increase a lot	66.9	72.1		69.7	71.1	
Public health education programs			0.007*			0.098
Not increase at all	13.1	7.1		11.2	7.6	
Increase a little	22.8	22.1		19.7	23.5	
Increase a lot	64.1	70.9		69.1	68.8	
Safer roads for public			0.061			0.470
Not increase at all	16.6	12.0		13.9	13.0	
Increase a little	21.7	19.0		17.6	20.8	
Increase a lot	61.7	69.0		68.5	66.1	
Programs to support the poor, e.g., housing			0.000*			0.000*
Not increase at all	15.2	6.9		11.8	8.1	
Increase a little	13.4	19.1		11.2	20.5	
Increase a lot	71.4	74.0		77.0	71.4	
Infrastructural development of the country			0.000*			0.102
Not increase at all	15.5	6.8		12.1	7.9	
Increase a little	15.9	20.7		18.5	19.6	
Increase a lot	68.6	72.6		69.4	72.4	
Total sample	290	707		330	667	

*p ≤ 0.05.

When controlled for confounding (Tables 9, 10) individuals aged 50–64 years are 40% less likely to agree that policy measures aimed at reducing alcohol consumption can benefit the public, regardless of whether they consume alcohol or not, compared to those aged 18–33 years. Similarly, individuals who consumed alcohol in the past 12 months and were exposed to media messages on alcohol are also less likely to agree with this statement compared to those who did not consume alcohol and were not exposed to such messages. However, men are 2.43 times more likely to agree with the statement as compared to women.

**Table 9:** Regression analysis on attitude toward alcohol, alcohol industry and governments policies toward alcohol.

Statements	Age of respondent				Gender		Education			Alcohol consumption in past 12 months		Exposure to media messages on alcohol at baseline	
	18–33 years	34–49 years	50–64 years	65+ years	Women	Men	No formal schooling or primary education	Studied or passed O/Ls	Studied or passed A/Ls or higher	No	Yes	Unaware	Aware
Support for government policies													
Policy measures to reduce the consumption of alcohol can benefit the public whether they consume alcohol or not. (agree)	Ref	0.90	0.61*	0.79	Ref	2.43**	Ref	1.09	0.59	Ref	0.45**	Ref	0.46**
Increasing the price of alcohol would help address the problem of alcohol consumption in Sri Lanka. (agree)	Ref	0.77	0.92	1.13	Ref	0.86	Ref	1.19	0.73	Ref	0.87	Ref	0.69*
Taxes on alcohol products are an effective way to reduce alcohol consumption. (agree)	Ref	0.98	1.28	0.81	Ref	0.93	Ref	1.47	0.87	Ref	1.05	Ref	0.89
Attitude toward government													
It is important for my government to be involved in solving the problem of alcohol consumption in my country. (agree)	Ref	1.09	1.98	1.23	Ref	1.44	Ref	1.26	0.98	Ref	0.63	Ref	0.80
The government’s alcohol laws are currently strong enough and protect people from alcohol harms. (agree)	Ref	1.23	1.47	2.32**	Ref	0.50**	Ref	2.37**	1.58	Ref	1.29	Ref	0.87
Attitude toward alcohol industry													
The alcohol industry is an obstacle to getting strong alcohol laws in the country. (agree)	Ref	0.98	1.05	1.04	Ref	0.90	Ref	1.46	1.30	Ref	0.89	Ref	0.76
Attitude toward alcohol													
Alcohol is an obstacle to development. (agree)	Ref	1.31	1.59*	3.01**	Ref	0.68	Ref	1.74*	1.74*	Ref	0.59**	Ref	1.02
Alcohol use can result in alcohol addiction. (agree)	Ref	0.99	1.26	4.85**	Ref	0.80	Ref	1.59	0.95	Ref	0.30**	Ref	1.06
Alcohol consumption affects others and the wider community negatively. (agree)	Ref	1.08	1.29	1.77	Ref	0.62	Ref	1.25	0.97	Ref	0.37**	Ref	1.30
Drinking alcohol, a couple of times a week is not harmful to health. (agree)	Ref	0.99	1.01	1.16	Ref	0.75	Ref	1.77*	1.98**	Ref	0.87	Ref	1.45*
Other statements													
Exposure to alcohol advertising encourages youth to initiate alcohol use. (agree)	Ref	1.02	1.66	2.69*	Ref	0.78	Ref	2.01*	1.49	Ref	0.49**	Ref	1.21

*Significant at 5%; **Significant at 1%. “Ref” indicates the reference category for that independent variable. Other response categories within the same variable are compared with the reference category.

**Table 10:** Regression analysis support for government policies related to alcohol.

	Age of respondent				Gender		Education			Alcohol consumption in past 12 months		Exposure to media messages on alcohol at baseline	
	18–33 years	34–49 years	50–64 years	65+ years	Women	Men	No formal schooling or primary education	Studied or passed O/Ls	Studied or passed A/Ls or higher	No	Yes	Unaware	Aware
To what extent, would you support the government in implementing below policies?													
Stop indirect/direct alcohol advertising and promotion on traditional media, like TV, radio and social media. (a great deal + very much)	Ref	1.41*	1.95**	0.82	Ref	1.90**	Ref	0.98	0.54*	Ref	0.49**	Ref	0.44**
Reduce the number of places and times where people can buy and consume alcohol. (a great deal + very much)	Ref	0.99	1.12	0.68	Ref	1.95**	Ref	0.45**	0.36**	Ref	0.30**	Ref	0.60**
Educate the population about alcohol harms. (a great deal + very much)	Ref	1.26	1.98**	0.87	Ref	1.69**	Ref	1.41	0.73	Ref	0.76	Ref	0.44**
Provide services for people affected by alcohol. (a great deal + very much)	Ref	1.23	1.36	0.88	Ref	1.54**	Ref	0.96	0.65	Ref	0.70*	Ref	0.49**
Raising the legal drinking age. (a great deal + very much)	Ref	1.17	1.66*	1.38	Ref	1.39*	Ref	0.86	0.37**	Ref	0.40**	Ref	0.60**
Any other recommendation for the government regarding controlling alcohol problems													
Cancelation of all alcohol licenses	Ref	1.2	1.47	1.3	Ref	1.33	Ref	1.18	0.87	Ref	0.53**	Ref	0.85
Stop manufacturing, importing, and selling alcohol	Ref	0.74	1.05	0.89	Ref	1.44*	Ref	0.88	0.73	Ref	0.59**	Ref	0.81
Strengthen the law enforcement in the country	Ref	1.09	1.05	1.10	Ref	0.83	Ref	0.67	1.07	Ref	1.06	Ref	1.65**
Stop issuing new license to sell alcohol	Ref	0.99	1.45	0.90	Ref	0.98	Ref	1.91	1.69	Ref	0.79	Ref	1.31
Stop all types of alcohol promotions	Ref	1.19	1.24	1.64	Ref	1.39	Ref	1.99	2.47*	Ref	0.78	Ref	0.86
Stop the alcohol industry’s influence on policy makers and politicians	Ref	0.84	0.99	2.80*	Ref	0.79	Ref	2.85	2.56	Ref	0.71	Ref	0.76
Stop giving bar license to politicians	Ref	1.22	1.85*	0.72	Ref	0.71	Ref	1.07	2.19	Ref	1.59	Ref	1.39
Cancel bar license owned by politicians	Ref	1.33	2.02*	1.47	Ref	0.72	Ref	1.43	3.27*	Ref	1.80*	Ref	1.30
Your support of the government proposal to tax alcohol products increase if the government committed to spending some of the revenue on the following public programs?													
Improve primary health care for all citizens (universal health care). (increase a lot)	Ref	1.20	1.01	0.80	Ref	1.28	Ref	1.02	0.89	Ref	0.88	Ref	0.72*
Primary school education (Increase a lot)	Ref	1.04	1.25	1.12	Ref	0.99	Ref	0.90	0.79	Ref	0.77	Ref	0.99
Public health education programs (Increase a lot)	Ref	1.09	0.91	0.86	Ref	1.004	Ref	0.75	0.65	Ref	0.71	Ref	1.08
Safer roads for public (Increase a lot)	Ref	1.06	0.92	1.35	Ref	1.31	Ref	0.84	0.95	Ref	0.63**	Ref	1.13
Programs to support the poor, e.g., housing (Increase a lot)	Ref	1.02	0.98	1.38	Ref	0.70*	Ref	0.84	0.59	Ref	0.97	Ref	1.40*
Infrastructural development of the country (Increase a lot)	Ref	0.99	0.96	1.48	Ref	1.00	Ref	1.17	1.06	Ref	0.82	Ref	0.87
Total sample	384	293	217	103	352	645	99	387	511	290	707	330	667

*Significant at 5%; **Significant at 1%. “Ref” indicates the reference category for that independent variable. Other response categories within the same variable are compared with the reference category.

Individuals exposed to media messages on alcohol are 31% less likely to agree with the statement “Increasing the price of alcohol would help address the problem of alcohol consumption in Sri Lanka” compared to those who were not exposed to such messages.

With increasing age and education, respondents tend to be more likely to agree that the government’s alcohol laws are currently strong enough and protect people from alcohol-related harms, with odds ratios of 2.32 for the 65+ years age group and individuals who have studied or passed O/Ls have an odds ratio of 2.37 compared to those with no formal schooling or primary education. However, men are 50% less likely than women to agree with this statement.

With increasing age, respondents tend to be more likely to agree that alcohol is an obstacle to development, with odds ratios of 1.59 for the 50–64 years’ age group and 3.01 for the 65+ years age group compared to the 18–33 age group. Moreover, individuals who have studied or passed O/Ls or studied or passed A/Ls or higher have an odds ratio of 1.74 compared to those with no formal schooling or primary education. However, individuals who consumed alcohol in the past 12 months are 40% less likely than those who did not to agree with this statement.

## 4 Discussion

This opinion survey was conducted to analyze attitude toward the government and alcohol industry, assess public support for alcohol control policies, and gage the level of support for implementing these policies in Sri Lanka.

The study reports a prevalence of 29.08% (*n* = 290) alcohol consumption, The periodic trend survey conducted by ADIC each year also report a rate of 25% in year 2022 and the STEPS survey (14) conducted by the Ministry of Health, Sri Lanka reports a prevalence of 20.7% for both sexes and 43.3% for males and 1.2% for females. Additionally, the high discrepancy between the two sexes is well observed in the present study and justifies the methodological decision on taking a 2:1 ratio sample.

The findings revealed that the majority of the study participants hold a favorable attitude toward alcohol control policy measures and support strengthening the regulatory framework for alcohol in the country. Therefore, this study highlights the importance of raising public awareness about the impact of alcohol policies designed to reduce alcohol consumption and related harm, which is crucial in fostering a public movement in both strengthening and enforcing alcohol control policy measures in the country.

Alcohol consumption shows a declining trend with increased education levels and differs according to the types of alcohol consumed. The results shows that arrack and beer are the most commonly consumed type of alcohol; beer is mostly consumed by younger age groups. Women only consumed beer and arrack. Consumption of beer increased, and toddy decreased with increased level of education. Moreover, toddy and kasippu showed significantly low levels of consumption among the study participants. These results are consistent with prior research and represents the current situation with regard to alcohol consumption patterns in Sri Lanka (14, 15).

The study shows that a majority hold the view that existing alcohol regulations lack the necessary strength to safeguard individuals from alcohol-related harm. Additionally, participants perceive that the alcohol industry is an obstacle to the establishment of strong alcohol laws in the country. A majority of study participants expressed the belief that existing alcohol laws lack sufficient strength to safeguard individuals from alcohol-related harm. Consequently, many participants emphasized the necessity for government intervention in addressing the issue of alcohol consumption within the country. A substantial majority of both female and male participants agreed with the statement that policy measures aimed at curbing alcohol consumption can yield public benefits.

Most of the participants agree that alcohol consumption can result in alcohol addiction and is an obstacle to development and affects others and the wider community negatively. The findings also revealed a notable level of public endorsement for alcohol control policy measures, indicating a favorable environment for establishing effective alcohol control policies within the country. In terms of specific government action with regard to alcohol control in the country, the majority supported increasing alcohol prices and alcohol taxation as beneficial measures to address the alcohol consumption problem in Sri Lanka.

A survey conducted in New South Wales, Australia reported contrasting results, indicating a low level of public support for measures such as price increases and taxation aimed at alcohol control (16). In this study, participants showed significant support for measures to reduce alcohol availability including reducing the number of outlets and times where alcohol can be bought and consumed, public education on alcohol harm, providing services for people affected by alcohol, and raising legal age for alcohol use. In another study exploring the public opinion in seven countries, restrictions on the number of alcohol outlets was one of the least favored policies among the study participants (17).

Many survey participants reported that exposure to alcohol advertising encourages youth to initiate alcohol use hence a high proportion of 68.4% indicated high level of support for the government to stop both direct and indirect promotions of alcohol through media platforms. This is in contrast with findings of a survey conducted across seven countries characterized by diverse sociocultural backgrounds, where the public support was significantly lower for alcohol advertising restrictions (17). Our study findings reveal that individuals who do not consume alcohol demonstrated a higher level of support for price increases on alcohol products compared to those who do consume alcohol.

Additional measures to reduce alcohol consumption, supported by survey participants, include enhancing law enforcement within the country for existing policies, halting the manufacturing, importing, and selling of alcohol, and revoking all alcohol licenses.

Participants indicated that their support for increasing taxes on alcohol products would significantly rise if a portion of the revenue generated from alcohol taxes were allocated to programs benefiting poor people (such as housing initiatives); infrastructure development; primary school education; public health education programs; universal healthcare; and the construction of safer roads for the public.

Therefore, these findings indicate that the public, regardless of their alcohol consumption status, prioritize societal well-being and overall development of the country, suggesting a collective concern for addressing these needs.

As these findings indicate a positive support for public health and alcohol policies in Sri Lanka, ADIC has leveraged these findings for lobbying and advocacy purposes in its interventions, mainly targeting key politicians, higher officials in the finance and health ministries, key government officials, key opinion leaders, civil society organizations, and media. These lobbying and advocacy efforts facilitated discussion about the level of understanding of the public on increasing alcohol tax and other important policies related to alcohol tax and described public opinion on alcohol harms.

## 5 Limitations of the study

The study was conducted as a street intercept survey, which comes with certain limitations. The lack of a confidential environment restricted our ability to inquire about sensitive data, such as drinking patterns and frequency. Additionally, this setting may have introduced volunteer bias, despite efforts by data collectors to randomly select participants. While questions regarding opinions were carefully constructed and asked in a non-leading manner, and the tool underwent pretesting, it is not a validated tool to capture public opinion on alcohol. Therefore, there may be a small degree of response and misclassification bias. The role of other possible confounders such as tobacco and illicit drugs were not explored due to feasibility issues.

This cross-sectional study aimed to maintain a 2:1 ratio of male to female respondents to capture the opinions of drinkers on alcohol policies. Given the significantly lower alcohol consumption among females in Sri Lanka, more males were interviewed for the survey. However, it’s crucial to acknowledge this limitation as it may impact the sample’s representativeness. Another limitation of the study is that the response rate was not calculated, potentially affecting the assessment of the study’s representativeness and generalizability of findings.

## 6 Conclusions and recommendations

Our work delves into public perceptions of alcohol-related policies in Sri Lanka, examining current trends in alcohol use, attitude toward alcohol consumption, government and public support for policies, and differences in support across sociodemographic groups and drinking statuses. Despite Sri Lanka’s effective COVID control, the economic crisis necessitates revenue increases, with alcohol tax hikes being a key consideration. However, policymakers’ divergent views, some fearing voter backlash, complicate matters. Our findings underscore public support for robust alcohol control policies, emphasizing awareness of government costs and the role of effective policy in addressing alcohol-related issues.

## Data availability statement

The raw data supporting the conclusions of this article will be made available by the authors, without undue reservation.

## Ethics statement

The data collection was conducted in accordance with the Declaration of Helsinki. Before initiating the interview, the study was explained to all the participants. Verbal consent was taken from each interviewee regarding their participation in the study. The questionnaire was administered only to those participants who agreed to participate. No personal identifiable information was obtained during the data collection.

## Author contributions

NC: Conceptualization, Methodology, Supervision, Writing – original draft, Writing – review & editing, Formal analysis, Project administration. NN: Formal analysis, Writing – review & editing, Methodology. HS: Funding acquisition, Project administration, Supervision, Writing – original draft. SS: Conceptualization, Funding acquisition, Methodology, Project administration, Resources, Validation, Writing – review & editing. NS: Project administration, Supervision, Writing – review & editing. KP: Project administration, Supervision, Writing – review & editing. RR-H: Formal analysis, Writing – review & editing. NM: Formal analysis, Writing – review & editing, Methodology.

## Footnotes

^1^A/L: G.C.E. Advanced Level entails 2 years of formal education in the school system following the completion of GCE O/L. A/L is a national level examination that students take at the end of secondary school in Sri Lanka, in grade 13, usually by students aged 18 years. Educational qualifications higher than A/L includes completing diploma or degree level qualifications. O/L: G.C.E Ordinary Level (O/L – once 11 years of formal school education is completed, students sit for the G.C.E O/L exam, which is a national level examination that students take typically at the age of 16 years, to become eligible to continue studies beyond this level).
