# Corrigendum: Public opinion on alcohol policies in Sri Lanka

**DOI:** 10.3389/fpubh.2024.1479396

**Published:** 2024-09-03

**Authors:** Nadeeka K. Chandraratne, Nalin Singh Negi, Hasini Siyambalapitiya, Sampath De Seram, Nidarshana Selladurai, Karieshini Pieris, Rachel Rothenstein-Henry, Nandita Murukutla

**Affiliations:** ^1^Faculty of Medicine, University of Colombo, Colombo, Sri Lanka; ^2^Vital Strategies, New Delhi, India; ^3^Alcohol and Drug Information Centre, Colombo, Sri Lanka; ^4^Vital Strategies, New York, NY, United States

**Keywords:** alcohol, public opinion, tax, policy, Sri Lanka

In the published article, there was a mistake in the Acknowledgement section. Tom Carroll, was not mentioned in the acknowledgements section.

The Acknowledgments section previously stated:

“Authors wish to acknowledge Pubudu Sumanasekara and Amaranath Tenna for their contribution in implementation of the project.”

The corrected Acknowledgment statement appears below:

“Authors wish to acknowledge Pubudu Sumanasekara and Amaranath Tenna for their contribution in implementation of the project and Tom Carroll for providing guidance in developing the questionnaire.”

The authors apologize for this mistake and state that this does not change the scientific conclusions of the article in any way. The original article has been updated.

